# Comparison of the ID Now Influenza A & B 2, Cobas Influenza A/B, and Xpert Xpress Flu Point-of-Care Nucleic Acid Amplification Tests for Influenza A/B Virus Detection in Children

**DOI:** 10.1128/JCM.01611-19

**Published:** 2020-02-24

**Authors:** Neena Kanwar, Jeffrey Michael, Kathryn Doran, Emily Montgomery, Rangaraj Selvarangan

**Affiliations:** a Children’s Mercy Hospital and Clinics, Kansas City, Missouri, USA; University of North Carolina School of Medicine

**Keywords:** children, influenza detection, molecular diagnostic assays performance

## Abstract

Early diagnosis of influenza (Flu) is critical for patient management and infection control. The ID Now influenza A & B 2 (ID Now) assay (Abbott Laboratories), Cobas influenza A/B nucleic acid test (LIAT; Roche Molecular Systems, Inc.), and Xpert Xpress Flu (Xpert; Cepheid) are rapid, point-of-care molecular assays for Flu virus detection. The study aim was to compare the performances of these three commercially available Clinical Laboratory Improvement Amendments (CLIA)-waived Flu virus assays. We prospectively enrolled 201 children <18 years old from January to April 2018 and collected nasopharyngeal swab specimens in viral medium. Aliquots were frozen for testing on different diagnostic platforms, as per the manufacturers’ instructions. CDC Flu A/B PCR was used as a reference method to evaluate the performances of these three platforms. Among the 201 specimens tested, the CDC Flu A/B PCR assay detected Flu A/B virus in 107 samples (Flu A virus, 73 samples; Flu B virus, 36 samples; dual Flu A/B virus positive, 2 samples), while the ID Now virus detected 102 samples (Flu A virus, 69 samples; Flu B virus, 37 samples; dual Flu A/B virus positive, 4 samples; invalid rate, 1/201 [0.5%]), the LIAT detected 112 samples (Flu A virus, 74 samples; Flu B virus, 38 samples; invalid rate, 11/201 [5.5%]), and the Xpert assay detected 112 samples (Flu A virus, 76 samples; Flu B virus, 36 samples; invalid rate, 6/201 [3.0%]). The overall sensitivities for the ID Now assay, LIAT, and Xpert assay for Flu A virus detection (93.2%, 100%, and 100%, respectively) and Flu B virus detection (97.2%, 94.4%, and 91.7%, respectively) were comparable. The specificity for Flu A and B virus detection by all methods was >97%. These molecular assays had higher sensitivity than did a historical standard-of-care test from the BD Veritor antigen test (Flu A virus, 79.5%; Flu B virus, 66.7%).

## Abstract (precis)

Early diagnosis of influenza (Flu) is critical for patient management and infection control. The ID Now influenza A & B 2 (ID Now) assay (Abbott Laboratories), Cobas influenza A/B nucleic acid test (LIAT; Roche Molecular Systems, Inc.), and Xpert Xpress Flu (Xpert; Cepheid) are rapid, point-of-care molecular assays for Flu virus detection. The study aim was to compare the performances of these three commercially available Clinical Laboratory Improvement Amendments (CLIA)-waived Flu virus assays.

## INTRODUCTION

Respiratory infection by influenza (Flu) virus is capable of causing severe illness resulting in hospitalization and death in young children and the elderly (1, 2). Early diagnosis of influenza virus is critical for patient management, infection control, and reduction of health care costs (3–5). Various influenza virus testing methods are available, including antigen detection-based assays (rapid influenza diagnostic assays [RIDTs] and immunofluorescence assays), molecular assays (rapid molecular assays, reverse transcription-PCR [RT-PCR], and other nucleic acid amplification assays), and viral culture (https://www.cdc.gov/flu/professionals/diagnosis/overview-testing-methods.htm). Sensitivities and specificities vary among the various methods currently approved for detecting Flu A and B viruses (4, 6). The list of Food and Drug Administration (FDA)-cleared molecular assays for influenza virus detection can be found at https://www.cdc.gov/flu/pdf/professionals/diagnosis/table1-molecular-assays.pdf. Molecular assays provide higher sensitivity and specificity than do RIDTs. Rapid, point-of-care molecular assays yield results that are available in approximately 30 min (7). The Infectious Diseases Society of America (IDSA) recommends using rapid influenza molecular assays over RIDTs for detecting influenza virus in specimens from suspected respiratory infection patients in the outpatient clinical setting (8).

The ID Now influenza A & B 2 (ID Now) assay (Abbott, IL; formerly known as Alere i influenza A/B 2), Cobas influenza A/B nucleic acid test (LIAT; Roche Molecular Systems, Inc., Indianapolis, IN), and Xpert Xpress Flu (Xpert) assay (Cepheid, Sunnydale, CA) are FDA-cleared, rapid, point-of-care detection systems for influenza virus. ID Now uses isothermal nucleic acid amplification technology based on nicking enzyme amplification reaction (NEAR) technology, while the LIAT and the Xpert assay are real-time RT-PCR assays. All three platforms use single-use test units containing target-specific reagents where the extraction, amplification, and detection of influenza A and B viral RNA take place. This research study was designed to compare the performance of these three Clinical Laboratory Improvement Amendments (CLIA)-waived Flu virus molecular detection systems (ID Now assay, LIAT, and Xpert assay) using nasopharyngeal swab specimens collected from children during the 2017–2018 respiratory season. The performance of each system was evaluated against the CDC Flu A/B PCR. The results from the BD Veritor Flu A/B antigen test (BD Diagnostics, Sparks, MD) were obtained from standard-of-care testing and compared with those from the three molecular assays.

## MATERIALS AND METHODS

### Study design and specimens.

This prospective clinical trial was conducted from January to April 2018 at Children’s Mercy Hospitals and Clinics in Kansas City, MO, USA, to evaluate the performances of three molecular assays (ID Now, LIAT, and Xpert) and an antigen-based BD assay. The study samples came from 201 male and female subjects between the ages of 0 and 200 months (median, 42 months) with suspected respiratory infections. Subjects with a physician’s order for a Flu test were eligible to participate in the study. After the standard-of-care test was completed, the subject was approached and enrolled in the study. Following parental permission and the child’s assent when appropriate, a nasopharyngeal swab specimen was obtained and saved in 3 ml of universal transport medium. Each specimen was given a unique study identifier at the time of study enrollment. Five aliquots of 300 μl were made and frozen at –70°C. Aliquots 1, 2, and 3 were thawed once before testing by the ID Now assay, LIAT, and Xpert assay at a later date. Aliquot 4 was thawed and extracted on the NucliSENS easyMAG instrument (bioMérieux, Inc., Durham, NC, USA). Nucleic acid aliquots were prepared for performing the CDC Flu A/B PCR in a batch mode. Aliquot 5 was kept as a backup aliquot for any repeat runs resulting from invalid results and also for running a subset of the samples on the early callout mode on the ID Now platform. BD Veritor antigen test results were obtained from standard-of-care testing; results for 200 subjects were available, with one of the subjects having a PCR-based assay ordered for standard-of-care testing. The results for all three molecular assays and the results from the BD antigen assay were compared with results from the reference FDA-cleared influenza PCR assay (CDC Flu A/B PCR). Positive and negative controls were run on a weekly basis on all three molecular assays. Invalid results were retested once.

### CDC Flu A/B PCR test.

Nucleic acid extraction was performed with the NucliSENS easyMAG system from a 200-μl sample (aliquot 4) containing 20 μl of universal internal control along with positive and negative extraction controls, according to the manufacturer’s instructions; the nucleic acid obtained was eluted in 110 μl of elution buffer. The sample eluate was aliquoted into two separate Eppendorf tubes and stored at −80°C until further testing. CDC Flu A/B PCR is a real-time RT-PCR-based assay that was performed on an Applied Biosystems 7500 Fast Dx system (Thermo Fisher Scientific Corp., MA, USA) with software version 1.4. The assay targets a region of the matrix gene of influenza A virus and a region of the nonstructural protein gene of influenza B virus. Oligonucleotide primers and probes for characterization and differentiation of influenza A subtypes (pandemic H1N1 [pH1N1] and H3N2 virus strains) and genetic lineages of influenza B virus (Yamagata and Victoria) target highly conserved regions of the hemagglutinin (HA) gene. The results from this assay were used for the sensitivity and specificity analyses.

### ID Now assay.

The ID Now assay is an isothermal nucleic acid amplification-based, sample-to-answer assay indicated for use on the ID Now platform for the detection and differentiation of influenza A virus and influenza B virus. The test targets polymerase basic gene 2 (PB2) for influenza A virus and polymerase acidic gene (PA) for influenza B virus detection. The test uses 200 μl of sample volume. Results are provided in approximately 15 min, including upfront 3 minutes of sample elution buffer warm-up time, followed by the assay run time. The early callout mode is an early detection feature of the new ID Now assay that allows the assay to end as soon as a positive result is detected in one of the targets (Flu A or Flu B virus). The assay run time on the ID Now platform was within 2 to 5 min for a positive call in the early callout mode as opposed to less than 13 min on the full-callout mode. The time taken for a negative result call on the early callout mode was similar to that with the full-callout mode. The warm-up time required for the sample elution buffer in the newer version is reduced by half, from 6 min to 3 min. The manufacturer’s instructions were followed to perform the assay; the detailed method can be found in an earlier publication (9). The performance characteristics presented in the manuscript were results from samples run on the full-callout mode. A subset of 102 samples was run on the early callout mode with aliquot 5 to determine the approximate time to result and agreement between the results from the two callout modes.

### Cobas influenza A/B nucleic acid test.

The Cobas influenza A/B nucleic acid test is a sample-to-answer system and real-time RT-PCR-based assay indicated for use on the Cobas LIAT PCR system (Roche Diagnostics, Indianapolis, IN). The assay targets a region of the matrix gene of influenza A virus and a region of the nonstructural protein gene of influenza B virus. The test uses 200 μl of sample volume, and results are provided in 20 min, with a hands-on time of less than 5 min. The manufacturer’s instructions were followed to perform the assay.

### Xpert Xpress Flu assay.

The Xpert Xpress Flu sample-to-answer assay is a real-time RT-PCR-based assay for the detection and differentiation of influenza A and B viral RNA. The samples were tested by the CLIA-waived assay on the Cepheid GeneXpert Xpress II system. The assay targets unique gene sequences that encode influenza A matrix (M), influenza A basic polymerase (PB2), and influenza A acidic (PA) proteins for influenza A virus and influenza B matrix (M) and influenza B nonstructural (NS) proteins for influenza B virus. The test was performed according to the manufacturer’s instructions using a 300-μl sample volume. Results were provided in approximately 30 min, with a hands-on time of less than 5 min.

### BD Veritor Flu A/B antigen test.

The BD Veritor Flu A/B antigen test is a chromatographic immunoassay and RIDT for qualitative detection of influenza A and B viral nucleoprotein antigens in respiratory specimens, with an instrument-based objective digital readout. The test uses a 300-μl sample volume. The results are obtained in less than 15 min. The test result from this standard-of-care assay was recorded and compared with the results from the molecular assays.

### Data analysis.

Overall Flu prevalence among the study participants was determined as per the CDC Flu A/B PCR assay (reference method). Age and symptom distribution among the Flu-positive and -negative groups were determined and statistically compared using Fisher’s exact two-tailed test. In addition, we fit a multivariable logistic regression model for examining the likelihood of testing positive for influenza in relation to clinical symptoms such as cough, sore throat, myalgia, conjunctivitis, and fever.

Two-by-two data tables were used to determine the sensitivity and specificity of all four Flu diagnostic assays in comparison with the reference method. The true-positive (TP), true-negative (TN), false-positive (FP), and false-negative (FN) categories were determined based on the reference method. Estimates along with 95% confidence intervals (CI) presented in Tables 2 and 3 were calculated from the site http://vassarstats.net/clin1.html.

## RESULTS

A total of 201 pediatric subjects with clinical symptoms suggestive of influenza infection were enrolled in this prospective study after informed consent was obtained. The median age for study subjects was 42 months (range, 0 to 200 months). Fever (96.5%), cough (86.6%), and fatigue (76.1%) were the most common symptoms observed in enrolled children. The presenting clinical symptoms that showed statistical significance (*P* < 0.05) between the Flu-positive and -negative groups by the CDC Flu A/B PCR assay were cough, fatigue, headache, myalgia, sore throat, and conjunctivitis. The multivariable logistic regression model revealed an increased likelihood of testing influenza positive when the patient presented with cough (adjusted odds ratio [aOR], 3.1), sore throat (aOR, 2.5), myalgia (aOR, 2.2), or conjunctivitis (aOR, 1.9). The number of days from onset of illness to date of enrollment for all 201 subjects and individual Flu-positive and -negative groups was similar (median [range], 1 [0 to 3] days). Detailed patient demographic and clinical information is listed in Table 1.

**TABLE 1:** Clinical characteristics overall and by Flu virus detection results as determined by the CDC Flu A/B PCR assay

Clinical characteristic	No. (%) in group:			*P* value
	Overall (*n* = 201)	Flu-positive result (*n* = 107)	Flu-negative result (*n* = 94)	
Fever	194 (96.5)	103 (96.3)	91 (96.8)	1.0
Cough	174 (86.6)	100 (93.5)	74 (78.7)	0.003
Fatigue	153 (76.1)	89 (83.2)	64 (68.1)	0.01
Headache	91 (45.3)	61 (57.0)	30 (31.9)	<0.001
Myalgias	84 (41.8)	57 (53.3)	27 (28.7)	<0.001
Sore throat	78 (38.8)	55 (51.4)	23 (24.5)	<0.001
Dyspnea	77 (38.3)	38 (35.5)	39 (41.5)	0.47
Abdominal pain	75 (37.3)	46 (43.0)	29 (30.9)	0.08
Conjunctivitis	72 (35.8)	47 (43.9)	25 (26.6)	0.01
Wheezing	70 (34.8)	36 (33.6)	34 (36.2)	0.77
Vomiting	56 (27.9)	30 (28.0)	26 (27.7)	1.0
Earache	53 (26.4)	24 (22.4)	29 (30.9)	0.2
Diarrhea	40 (19.9)	19 (17.8)	21 (22.3)	0.48

### Influenza virus.

The CDC Flu A/B PCR assay detected 73 Flu A and 36 Flu B cases among the 201 tested samples. The median (range) threshold cycle (*C_T_*) values for Flu A virus and Flu B virus were 23.3 (15.9 to 38.5) and 22.9 (16.9 to 36.5), respectively. Two samples were positive for both the Flu A and Flu B viruses. Subtyping of the 73 Flu A virus specimens with H1N1- and H3N2-specific primers detected 31 pH1N1 and 40 H3N2 strains. Two Flu A virus-positive strains remained untyped. Of the 36 Flu B virus specimens, 33 belonged to the Yamagata lineage, and the remaining 3 specimens could not be typed. We did not find any Victoria lineage strains among these samples.

### Flu A virus.

The three molecular platforms, ID Now, LIAT, and Xpert, were positive for 69, 74, and 76 samples, respectively, with a sensitivity of 100% for the LIAT and the Xpert assay and 93.2% for the ID Now assay. All five false-negative samples for the ID Now assay belonged to the pH1N1 subtype and had a high median *C_T_* value of 33.81 (range, 31.8 to 36.0) by the CDC Flu A/B PCR assay, suggesting low viral load. The antigen-based BD assay demonstrated a lower sensitivity of 79.5%, with overall 59 positive Flu A virus detections and 15 FN results compared with the CDC Flu A/B PCR assay. The median *C_T_* value for these 15 FN samples was 29.04 (range, 19.12 to 39.86) by the CDC Flu A/B PCR method. The specificity for the ID Now assay, LIAT, and BD assay was greater than 99%; the Xpert assay demonstrated a specificity of 97.7%, with three FP results. Table 2 depicts the details of all performance parameters.

**TABLE 2:** Performances of the ID Now, LIAT, Xpert, and BD assays versus that of the CDC Flu A/B PCR assay

Assay	Virus target	No. with result^*a*^:				% (95% CI) for:	
		TP	FP	TN	FN	Sensitivity	Specificity
ID Now	Flu A	68	1	127	5	93.2 (84.1–97.5)	99.2 (95.1–100.0)
	Flu B	35	2	163	1	97.2 (83.8–99.9)	98.8 (95.2–99.8)
LIAT	Flu A	73	1	127	0	100 (93.8–100)	99.2 (95.1–100.0)
	Flu B	34	4	161	2	94.4 (80.0–99.0)	97.6 (93.5–99.2)
Xpert	Flu A	73	3	125	0	100 (93.8–100)	97.7 (92.8–99.4)
	Flu B	33	3	162	3	91.7 (76.4–97.8)	98.2 (94.4–99.5)
BD^*b*^	Flu A	58	1	126	15	79.5 (68.1–87.7)	99.2 (95.0–99.9)
	Flu B	24	1	163	12	66.7 (48.9–80.9)	99.4 (96.1–99.9)

a TP, true positive; FP, false positive; TN, true negative; FN, false negative. The number of dual Flu A/B-positive results were 2 for the CDC Flu A/B PCR, 4 for the ID Now assay, 0 for the LIAT, 0 for the Xpert assay, and 0 for the BD assay.

b BD results were available for 200 subjects. Standard-of-care testing for one subject was PCR based.

### Flu B virus.

Among the three molecular assays, ID Now showed the highest sensitivity of 97.2%, followed by LIAT and Xpert, with sensitivities of 94.4% and 91.7%, respectively. The specificities of all three molecular assays were greater than 97%. The LIAT had four FP Flu B virus results, followed by the Xpert (*n* = 3) and ID Now (*n* = 2) assays. Of the 36 Flu B specimens detected by the CDC Flu A/B PCR assay, BD detected only 24 positives, with a sensitivity of 66.7%. The 12 FN samples had a median *C_T_* value of 28.1, ranging from 16.85 to 36.46. The details of all performance parameters are listed in Table 2.

### Invalid results.

Sample results were considered invalid if an instrument error was obtained or there was a failure to generate a result. The number of invalid results was highest for the LIAT (*n* = 11 [5.5%]), followed by Xpert (*n* = 6 [3.0%]) and ID Now (*n* = 1 [0.5%]). Repeat testing resulted in valid results for all affected samples. The rate of invalid runs for BD was undetermined since historic data were used for this platform.

### Early versus full-callout mode of the ID Now assay.

A total of 102 samples were run on both the early and full-callout modes. The time for a positive call on the early callout mode was less than 9 min, including the sample setup time. The assay run time for Flu B-positive results was observed to be faster than for Flu A-positive results (median [range], 3 [2 to 4] min for Flu B virus versus 4 [3 to 5] min for Flu A virus). The negative samples underwent a full cycle of approximately 13 min before the negative result call. The performances of the assay on the two callout modes were found to be comparable as well. The sensitivities for Flu A/B virus detection on both modes were found to be 95.8% and 100%, respectively. The specificity for Flu A virus detection was 100% for both modes, but the specificity for Flu B virus detection on the early callout mode was slightly lower than that on the full-callout mode (95.1% versus 97.5%, respectively). Detailed performance characteristics for both modes are given in Table 3.

**TABLE 3:** Performance characteristics of the ID Now assay on full- versus early callout mode

ID Now assay callout mode	Virus target	No. with result^*a*^:				% (95% CI) for:	
		TP	TN	FP	FN	Sensitivity	Specificity
Full	Flu A	23	78	0	1	95.8 (76.9–99.8)	100 (94.2–100)
	Flu B	21	79	2	0	100 (80.8–100)	97.5 (90.5–99.6)
Early	Flu A	23	78	0	1	95.8 (76.9–99.8)	100 (94.2–100)
	Flu B	21	77	4	0	100 (80.8–100)	95.1 (87.2–98.4)

a TP, true positive; FP, false positive; TN, true negative; FN, false negative.

## DISCUSSION

CLIA-waived rapid molecular assays are widely used as point-of-care assays for Flu virus detection in the clinical setting due to their high sensitivity and specificity, ease of use, and short turnaround time. The ID Now (Abbott) influenza A & B 2 assay, formerly the Alere i influenza A/B 2 assay, is the latest improved version of the Alere i influenza A/B assay. The ID Now influenza A & B 2 assay has incorporated significant changes to both the amplification reaction and the analytical software compared to the original Alere i influenza A/B assay. In this prospective study, we compared the performance characteristics of three CLIA-waived molecular platforms, LIAT, ID Now, and Xpert, for Flu virus detection in a pediatric population compared with the CDC Flu A/B PCR. BD results from standard-of-care testing were also used to compare the performances of the molecular detection platforms with this RIDT platform. The sensitivities for Flu A virus/Flu B virus (respectively) detection for the assays were as follows: 93.2%/97.2% for the ID Now assay, 100%/94.4% for the LIAT; 100%/91.7% for the Xpert assay, and 79.5%/66.7% for the BD assay. The specificities for the detection of both Flu A and B viruses were found to be greater than 97% for all assays. The invalid rate was lowest for the ID Now assay (0.5%) compared with the LIAT (5.5%) and the Xpert assay (3%). The time to result on the new ID Now platform for a positive sample was found to be less than 9 min in the early test mode, with performance similar to that of the full-callout mode.

The BD assay had lower sensitivity for both Flu A virus (79.5%) and Flu B virus (66.7%) than did all three molecular assays. The specificity of BD was comparable to that of the molecular assays. According to a recent meta-analysis (7) that included six studies evaluating the BD assay for Flu virus detection, sensitivity ranged from 64% to 94% for both Flu A and B virus detection. The difference in sensitivity is well acknowledged between an RIDT and a molecular assay. The same meta-analysis study (7) included seven studies for the Alere i assay and five studies for the LIAT. Although the pooled specificities for both assays for Flu A and B virus detection were found to be comparable (approximately 99%), a 12.4% difference in pooled Flu A virus detection sensitivity (Alere i, 84.4%; versus LIAT, 97.1%) and an 11.8% difference in Flu B virus detection sensitivity (Alere i, 86.6%; versus LIAT, 98.7%) were noted. The meta-analysis included studies from both the pediatric and adult populations. The new version of the ID Now assay appears to have significant improvement in Flu A and B virus detection, with Flu A virus sensitivity of 93.2% and Flu B virus sensitivity of 97.2%. The sensitivity for Flu B virus detection for the ID Now assay was found to be greater than for the LIAT (97.2% versus 94.4%, respectively).

Several studies have evaluated the performances of the Alere i influenza A & B, LIAT, and Xpert assays. The reported sensitivity for the Alere i assay for Flu B virus detection has ranged from 45.2% to 100% (9–15). The newer version evaluated in this study (ID Now) seemed to have improved sensitivity and specificity for both Flu A and Flu B virus detection. The performance parameters were found to be slightly higher than those of the other two molecular assays for Flu B virus detection. Among the 201 samples we tested in this study, only two (0.9%) samples were Flu B virus false-positive samples. These two FP samples for Flu B virus were also positive for Flu A virus by the ID Now assay. A higher number of FP results for Flu B virus was observed with the LIAT (*n* = 3 [1.5%]) and Xpert (*n* = 4 [2.0%]). The FP Flu B virus detection rate was comparable to the Flu B virus false results observed for the LIAT and the Xpert assay in our study.

Early Flu diagnosis is critical for patient management and reduction of morbidity and mortality during seasonal epidemics and pandemics (16). The assay processing time for the ID Now assay (approximately 13 min on the full-callout mode and less than 5 min on the early callout mode for a positive result) was faster than for the LIAT (20 min) and Xpert assay (30 min). Specimen processing for the ID Now assay was straightforward and took approximately 5 min in a few simple steps (the basic components of the test are the same as those of the previous versions; however, in the new version, the warm-up time required for the sample elution buffer is reduced by half, from 6 min to 3 min for both universal transport medium [UTM] and swab samples). The LIAT and the Xpert assay only required adding the sample to the cartridge. Other considerations for the point-of-care assay include an additional testing menu to limit multiple testing platforms, throughput, device footprint, sample volume, capability to interface with the laboratory information systems (LIS), storage space, and temperature conditions for the test devices. The three assays were comparable for most of these variables. GeneXpert systems that are suitable for most point-of-care settings are available in one-, two-, or four-module configurations. The Cepheid GeneXpert Xpress II system was used for this study. This instrument has the capacity to process two samples simultaneously and has random-access capability. The LIAT and the ID Now assay can handle only one sample at a time, but multiple instruments can be used to supplement additional testing. The linear square foot requirement was relatively greater for the Xpert II system (approximately 0.5 square feet) than with the ID Now assay (approximately 0.4 square feet) and the LIAT (approximately 0.3 square feet). In addition to the equipment, an additional benchtop area for a laptop may also be a consideration for Xpert. The LIAT required the kits to be stored at refrigeration temperatures, as opposed to the ID Now and Xpert assays, where the test kits could be stored at room temperature. Moreover, with the <5-min turnaround time with the ID Now early callout mode, results could be provided to the physician quickly, aiding in timely initiation of antiviral therapy and reduced health care costs, making it more suitable for the outpatient setting as a point-of-care assay.

The invalid rate is another important consideration when selecting an assay for clinical use. The invalid rate for the ID Now assay was significantly lower than that for the LIAT (0.5% versus 5.5%, respectively; *P* = 0.006) or the Xpert assay (0.5% versus 3%, respectively; *P* = 0.12), but the difference did not reach statistical significance. The retests performed on all assays yielded valid results. The invalid rate not only could add to laboratory costs but also cause a delay in reporting results. Earlier studies with the Alere i version reported invalid rates ranging from 0.5% to 4.2% (11–13, 17); there may be an improvement in the invalid rate as well with the current ID Now assay.

Strengths of our study include the prospective study design in which the subjects were enrolled systematically and a single NP swab specimen obtained to evaluate the performances of all 3 molecular assays. Study limitations include the fact that all three CLIA-waived molecular assays were evaluated by laboratory personnel and not in the point-of-care setting. Performance characteristics may differ when assays are performed by laboratory personnel rather than by clinical personnel in the point-of-care setting (18).

Overall, the performances of all three molecular assays were found to be comparable. ID Now is a CLIA-waived, simple-to-use molecular assay in which positive results can be obtained within 5 min. This advantage makes the assay suitable for point-of-care testing in an outpatient setting.
